# Experimental Study on Laser-Controlled Explosive Welding of Microscale Metallic Foils Driven by Energetic Materials

**DOI:** 10.3390/ma19030527

**Published:** 2026-01-28

**Authors:** Xiaojun Ye, Dongxian Ye, Yanshu Fu, Penglong Zhao, Xianfeng Xiao, Daomin Shi, Rui Zhang

**Affiliations:** 1 School of Information and Artificial Intelligence, Nanchang Institute of Science and Technology, Nanchang 330108, China; yexiaojun0512@126.com (X.Y.); yedongxian2@163.com (D.Y.); 2 School of Aeronautics Mechanical and Electrical Engineering, Jiangxi Flight University, Nanchang 330031, China; yshfu@jxfu.edu.cn (Y.F.); ncuzpl@163.com (P.Z.); zhangrui@jxfu.edu.cn (R.Z.); 3 School of Advanced Manufacture, Nanchang University, Nanchang 330031, China; xxf@ncu.edu.cn; 4 Tai’an Special Equipment Inspection and Research Institute, Tai’an 271000, China

**Keywords:** laser manipulated, microscale foils, precise EXW, packaging

## Abstract

In response to the challenge of achieving highly reliable interface fabrication in the fields of microelectronics and micro-electromechanical system (MEMS) packaging, this study harnesses the superior characteristics of solid-state bonding inherent in explosive welding (EXW) technology. This study investigates the precise EXW of milligram-scale metallic foils by employing focused laser energy to control the explosion behavior of liquid energetic materials, thereby generating shockwaves that induce high-velocity oblique collisions between metallic foils and base plates. Laser-focused energy was utilized to regulate energetic materials for conducting precision EXW experiments on Al/Cu couples. The technical feasibility and interfacial quality of this method for fabricating Al/Cu bonding interfaces were systematically evaluated through in situ observation of the dynamic welding process, comprehensive analysis of interfacial microstructures, and numerical simulations. The results reveal distinct Al/Cu elemental diffusion at the bonding interface, confirming the technical viability of the approach. However, an unloading rebound phenomenon is observed at the interface, which is inherently associated with the dynamic impact process, indicating the need for further optimization in the precise control of impact loading.

## 1. Introduction

Conventional explosive welding (EXW) utilizes the work done by detonation products of explosives to drive a flyer plate to collide with a base plate, forming a weld. It enables large-area metal plate cladding on a square-meter scale and is widely used in engineering structural components for automobiles, ships, the nuclear industry [1], petrochemical pipelines [2], and power transmission [3] due to its ability to achieve high-specific-strength and high-corrosion-resistance cladding of dissimilar metal pairs.

In EXW, the uniformity of thickness and density is difficult to achieve when using granular or powdered explosives due to inconsistencies in their placement, which limits the applicability of this technique to large-scale structural components in field welding applications. The poor controllability renders it unsuitable for welding metal foils thinner than 1 mm [4] and hinders the development of stable industrial production processes. Laser impact welding (LIW) is a process that uses laser energy to ablate a liquid medium coated on the flyer surface, causing rapid vaporization and phase explosion (not chemical explosion of energetic materials) to generate a shockwave that accelerates the foil for collision with the substrate. It can achieve bonding on a millimeter scale or even smaller areas, making it suitable for electronic integrated packaging in MEMS manufacturing. LIW technology was developed by Daehn [5] in 2011 and patented in the USA, extending EXW technology to micro- and fine-scale applications, and is now widely used in MEMS packaging. Reference [6] reported Al-Al and Al-steel cladding with a 3 mm diameter using a laser pulse with 8 ns duration and 3 J energy. A team from Jiangsu University in China [7] conducted research on Cu/Al welding with a 5 mm diameter based on this technology.

These two welding technologies are complementary not only in cladding size but also in their underlying working principle, which involves shockwave-driven, high-velocity oblique collision between metal plates, resulting in jetting that removes surface contaminants and enables metallurgical bonding under high pressure. Therefore, they are collectively classified as EXW. Moreover, the inherent precision and controllability of LIW offers a promising solution to the challenges associated with energy regulation in EXW. Current research on LIW energy regulation primarily focuses on laser input parameters, mostly exploring micro-welding effects by adjusting laser power, pulse width, and angle. Representative domestic work includes that conducted by the PLA Academy of Equipment [8,9,10], which indicated that the splashing phenomenon formed during laser ablation of liquid polymers is closely related to propulsion performance, with numerous controlling factors such as laser intensity, doping fraction, and solution viscosity. Current conclusions still provide a superficial understanding of the energy regulation mechanism. In particular, typical liquid polymer working media exhibit low absorption coefficients for infrared lasers, making them highly transparent to such radiation. To enhance laser absorption, infrared dyes or carbon powder are often added in practice, leading to dispersed regulation effects and consequently reduced precision [11]. Although LIW enables rapid small-scale bonding, it is challenging to control the consistency of the ablation phase change, often causing excessive pressure damage at the center of the welding interface and insufficient pressure leading to detachment at the edges [12].

Therefore, this study aims to investigate laser-controlled EXW of microscale foils driven by energetic materials. Unlike conventional LIW, which relies on laser ablation of inert polymers, the present method employs laser-initiated explosive reactions in energetic materials, offering enhanced controllability and more uniform interfacial bonding for microscale applications. Furthermore, numerical simulations based on the Smoothed Particle Hydrodynamics (SPH) method are employed to elucidate the dynamic welding process and interfacial formation mechanisms.

The working principle can be summarized as follows: first, a liquid energetic working medium is uniformly coated on the upper surface of the foil. Given the extremely low areal mass and structural stiffness of micro- and nanoscale foils, the driving energy required for collision welding is relatively small, corresponding to a coating thickness of liquid energetic material typically below its critical detonation dimension. Subsequently, focused laser energy is delivered to the liquid energetic medium, initiating an explosive reaction through localized energy deposition and generating a shockwave that drives the foil toward the substrate for precision EXW.

## 2. Experimental Principle and Scheme Design

The laser ignition process of energetic materials is essentially an interaction process between the laser and matter. According to the different mechanisms initiating the reaction in energetic materials, laser ignition mechanisms include thermal, photochemical, ionization, and shock initiation mechanisms. This paper focuses on utilizing the laser ignition method of energetic materials to explore precision welding of microscale foils. Its controllability is reflected in: (1) the uniformity of the thickness and compactness of the liquid energetic working medium coating is easily ensured; (2) since the coating thickness of the liquid energetic working medium is less than its critical detonation size, the explosive reaction cannot be self-sustaining and must be triggered by focused laser energy compensation. This allows the welding path to be determined by the laser movement path, and the welding interface size can be regulated by the characteristic scale of the laser focus.

### 2.1. Introduction to the Experimental System

**Figure 1:**
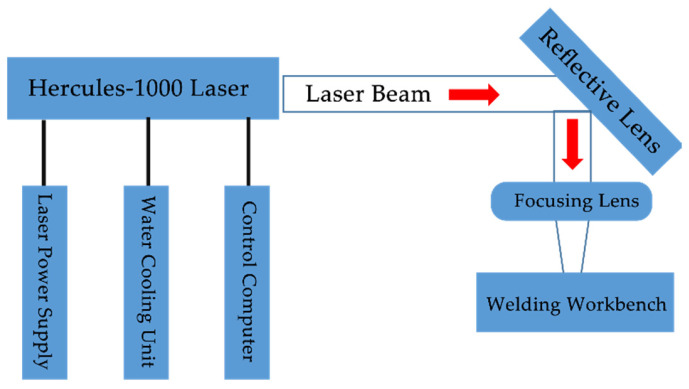
Welding experiment system.

**Figure 2:**
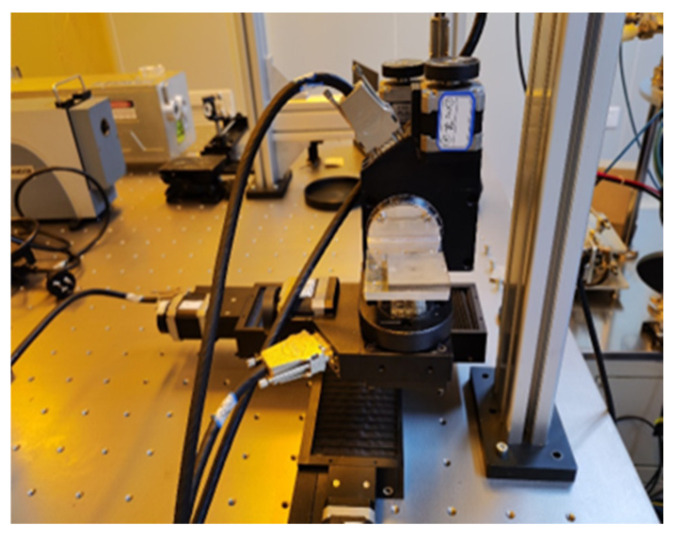
Laser controlled EXW platform.

**Figure 3:**
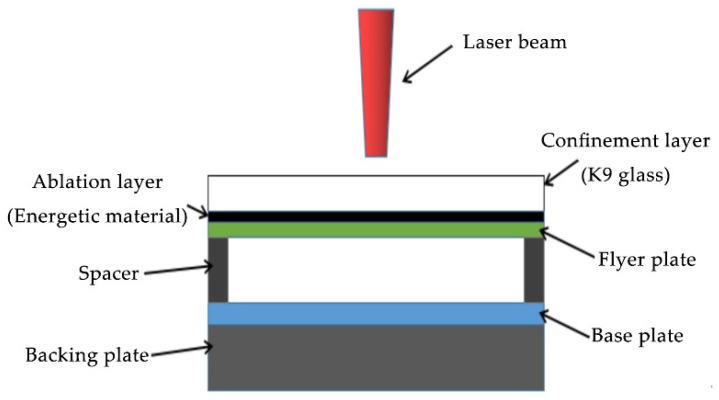
Welding device.

The welding experimental system is shown in Figure 1, primarily consisting of a laser emission device, laser power supply, water cooling device, computer control device, reflector, focusing lens, welding device, and welding platform. The laser used in this experiment is a lamp-pumped high-energy electro-optical Q-switched laser (Hercules-1000) produced by Anshan Ziyu Laser Technology Co., Ltd (Anshan, China). The laser device and welding platform are shown in Figure 2 and Figure 3, respectively. The main technical parameters of the Hercules-1000 laser are listed in Table 1.

The welding device is shown in Figure 3. From top to bottom, the components are arranged as follows: laser beam, confinement layer, ablation layer, flyer plate, spacer, base plate, and support plate. At the beginning of the welding process, the Gaussian laser beam irradiates the ablation layer through the glass window of the confinement layer. The energetic material in the ablation layer absorbs laser energy and is instantly converted into high-temperature plasma. However, due to the presence of the upper confinement layer, the high-temperature plasma can only impact the flyer plate downwards. The flyer plate is propelled downwards under the action of the high-temperature plasma, colliding with the base plate at high velocity to achieve welding.

### 2.2. Experimental Materials and Preparation

The materials required for this experiment and the preparatory work are as follows:

(1) Flyer and base plate materials: Industrial pure aluminum with a thickness of 50 μm and dimensions of 20 mm × 20 mm was selected as the flyer plate. T2 copper with a thickness of 100 μm and dimensions of 25 mm × 8 mm was selected as the base plate;
(2) Confinement layer material: K9 glass with a thickness of 6 mm, dimensions of 40 mm × 40 mm, and a central ground circular recess with a depth of 0.1 mm and a diameter of 5 mm;
(3) Energetic materials (ablation layer): Black paint, black tape, and diesel-ammonium nitrate gel were selected. The thickness of ablation layer was controlled to approximately 50 μm using the spin-coating technique to ensure uniformity and reproducibility. Surface morphology and elemental distribution were analyzed using scanning electron microscopy (SEM) coupled with energy-dispersive X-ray spectroscopy (EDX).

### 2.3. Experimental Parameter Design

During the experiment, the distance between the moving work platform and the focusing lens was controlled via the computer to achieve a laser spot diameter of 1.5 mm. By adjusting the laser settings, the laser energy was regulated at 717 mJ, 920 mJ, and 1280 mJ. Specific experimental parameters are provided in Table 2.

## 3. Experimental Results and Analysis

**Figure 4:**
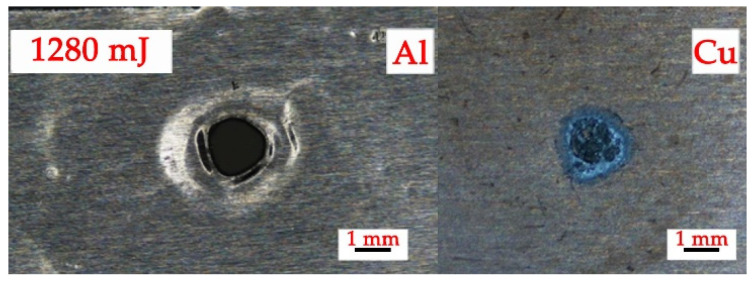
Surface morphology of blank assembly weldment.

The welding experiment results under different ablation layer materials and laser energies are shown in Table 3. According to the experimental results shown in Table 3, the surface morphology of the blank group specimen without energetic material coating is shown in Figure 4. Welding was not achieved, the Al foil was pierced, and Al ablation residue was observed on the surface of the copper base plate.

**Figure 5:**
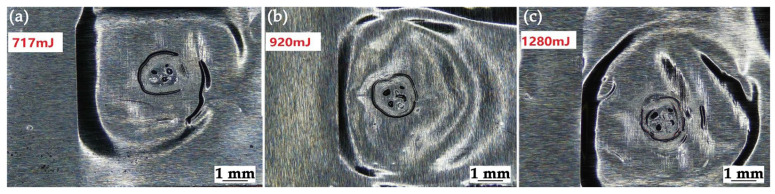
Surface morphology of the weldment under laser energies of (**a**) 717 mJ, (**b**) 920 mJ, and (**c**) 1280 mJ with a black paint ablation layer.

Table 3 summarizes the welding results under different ablation layer materials and laser energies. When the ablation layer material was black paint, successful welding was achieved at laser energy levels of 717 mJ and 920 mJ. In contrast, when diesel-ammonium nitrate gel was used as the ablation layer, welding success was observed only at a laser energy of 1280 mJ. This indicates that both the energy threshold and the ablation material’s absorption properties are critical for initiating the explosive reaction and generating sufficient shockwave pressure. The surface of the successfully welded specimen is shown in Figure 5. The bonding area is located within the red markings, with a range consistent with the spot size, approximately 1.5 mm in diameter. Simultaneously, many wrinkles and ripples can be observed in the welded zone. This is because the shockwave generated during welding propagates outward from the weld point, undergoing continuous reflection and refraction at the interfaces of the base and flyer plates. These wave interactions induce plastic deformation on the surface of the flyer metal, ultimately leading to the formation of ripples and wrinkles within the effective welding zone.

**Figure 6:**
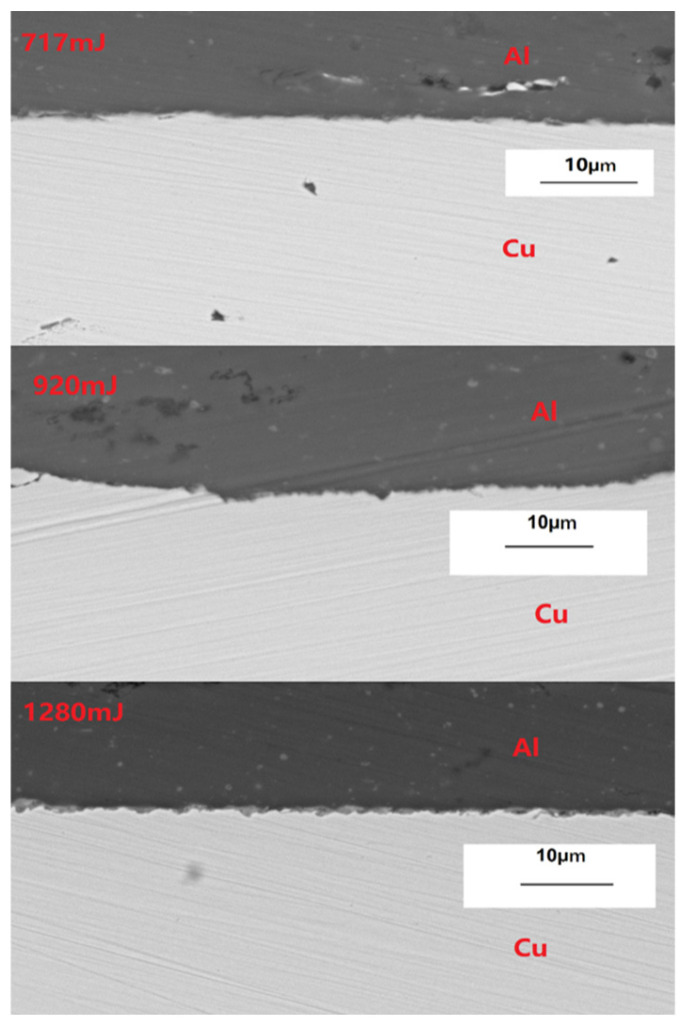
Microscopic morphology of laser energy-controlled welding interface in series.

Figure 6 shows the bonding interface morphology of welded specimens at laser energies of 717 mJ, 920 mJ, and 1280 mJ. It can be observed that when the laser energy is 717 mJ, the interface between the flyer and base plate is straight. When the laser energy increases to 920 mJ, the interface exhibits a slight wavy pattern. When the laser energy is 1280 mJ, the interface shows a distinct wavy pattern, with both wavelength and amplitude larger than those at 920 mJ. This is because increasing laser energy leads to greater kinetic energy conversion for the flyer plate, resulting in higher impact velocity against the base plate. This intensifies the degree of plastic deformation during collision bonding, consequently forming more pronounced wavy interfaces.

**Figure 7:**
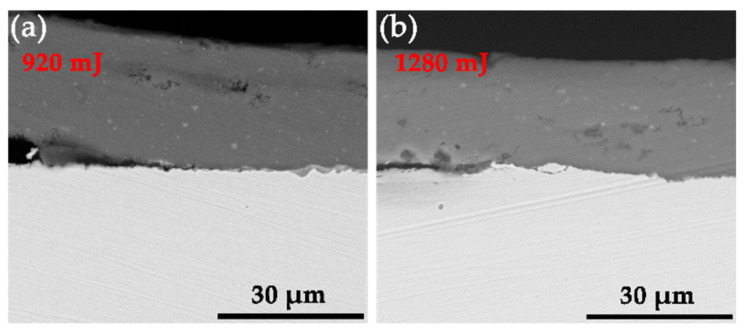
Interface characteristics of the cracking zone corresponding to laser energies of (**a**) 920 mJ and (**b**) 1280 mJ.

Figure 7 shows the characteristic morphology of the interface cracking zone corresponding to the series of laser-controlled energies. The cracking zone is located between the welded zone and the rebound zone and is directly connected to the welded zone. The formation mechanism of the cracking zone is as follows: When the flyer plate first contacts and collides with the base plate, the collision angle evolves from an initial value near 0°. At the onset of impact, the angle does not satisfy the weldable condition. As the collision proceeds and the angle enters the critical range required for welding, bonding initiates. However, due to the subsequent rebound phenomenon, part of the initially formed weld interface is torn apart by the expanding rebound zone, resulting in the formation of the cracking zone.

Preliminary EDS analysis confirmed elemental interdiffusion of Al and Cu at the bonded interface, suggesting metallurgical bonding. Future work will include systematic tensile shear testing, microhardness mapping, and ultrasonic inspection to quantitatively evaluate the mechanical integrity and bonding strength of the welded joints.

The observed rebound and cracking phenomena highlight the need for precise control of impact velocity and collision angle. Optimizing laser pulse shaping and confinement layer design may mitigate these effects, improving bonding continuity and interfacial strength.

## 4. Model and Material Parameters

### 4.1. Numerical Simulation Model Establishment

The SPH method, implemented within ANSYS AUTODYN 2019, was employed for the numerical simulations. The system of governing equations included the conservation of mass, momentum, and energy, integrated with the Johnson–Cook constitutive model to account for strain-rate and thermal softening. SPH, as a mesh-free Lagrangian technique, is well-suited for simulating extreme deformation and high-strain-rate events, including impact and explosion welding.

**Figure 8:**
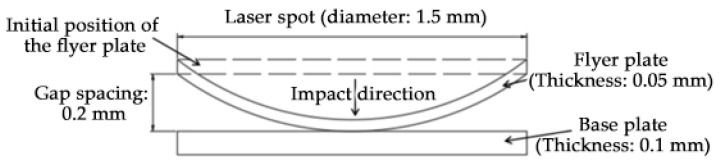
Numerical simulation model diagram of high-speed impact welding.

The pressure of the plasma shockwave induced by a short laser pulse exhibits a Gaussian distribution. Acting on the flyer plate, the flyer undergoes plastic deformation approximating a mushroom-shaped bulge before colliding with the base plate at several hundred meters per second [13]. Therefore, when establishing the model, the flyer was designed as an ideal arc, leading to the establishment of a two-dimensional plane model for high-speed impact welding of the flyer and base plate, as shown in Figure 8.

In the Al/Cu welding numerical simulation, the thickness of the Al flyer is 0.05 mm and the Cu base plate is 0.1 mm. The size of the SPH particles was set to 1 μm. The variable parameter in this simulation was the impact velocity of the flyer plate, with the initial loading condition defined as the flyer’s initial impact velocity. Based on previous related studies [14,15], the initial impact velocities of the flyer were set to 400 m/s, 600 m/s, and 800 m/s, respectively, while the initial velocity of the base plate was fixed at 0 m/s.

### 4.2. Welding Process

**Figure 9:**
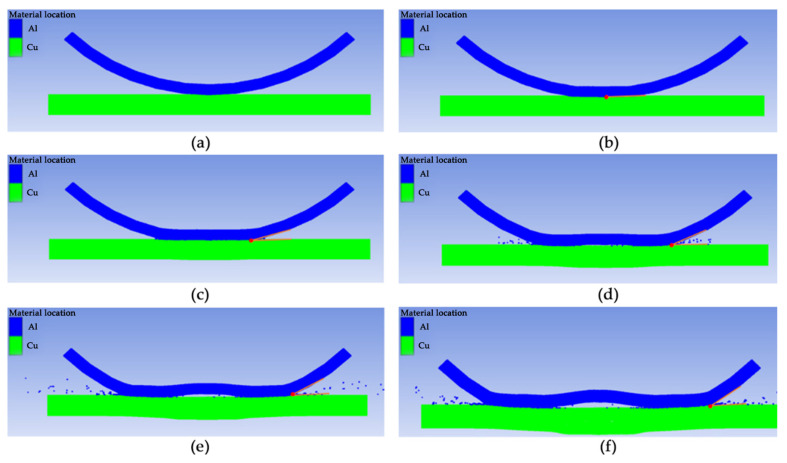
Welding process simulated using ANSYS AUTODYN (SPH solver): (**a**) 0 ns, (**b**) 20 ns, (**c**) 40 ns, (**d**) 90 ns, (**e**) 160 ns, (**f**) 200 ns.

Figure 9 presents the state diagrams of the Al/Cu high-speed impact welding process at different time intervals. Figure 9a illustrates the state at the initial moment of collision between the flyer and base plate when welding commences. Figure 9b depicts the condition at 20 ns after the onset of collision. At this stage, the collision angle β between the flyer and base plate is 3°, which is insufficient to satisfy the condition for jet formation; consequently, no jetting occurs. Figure 9c shows the state at 40 ns after the onset of collision. As the collision point progresses, the collision angle β increases to 12°, jetting begins to generate, and welding between the flyer and base plate commences. Figure 9d depicts the state at 90 ns after the onset of collision. The collision angle β continuously increases to 18°, substantial jetting occurs, and a rebound phenomenon begins to appear in the central area of the flyer. Figure 9e depicts the state at 160 ns after the onset of collision. The collision angle β has expanded to 25°, jetting continues to be ejected, and the flyer rebound phenomenon becomes increasingly pronounced. Figure 9f illustrates the state at 200 ns after the onset of collision. The collision angle β reaches 35°, the welding process is essentially complete, and a well-defined interfacial wave structure is clearly visible along the weld interface. The left and right regions correspond to bonded zones, whereas the central region forms the rebound zone, which is consistent with experimental observations.

### 4.3. Wavy Interface Morphology Characteristics

**Figure 10:**
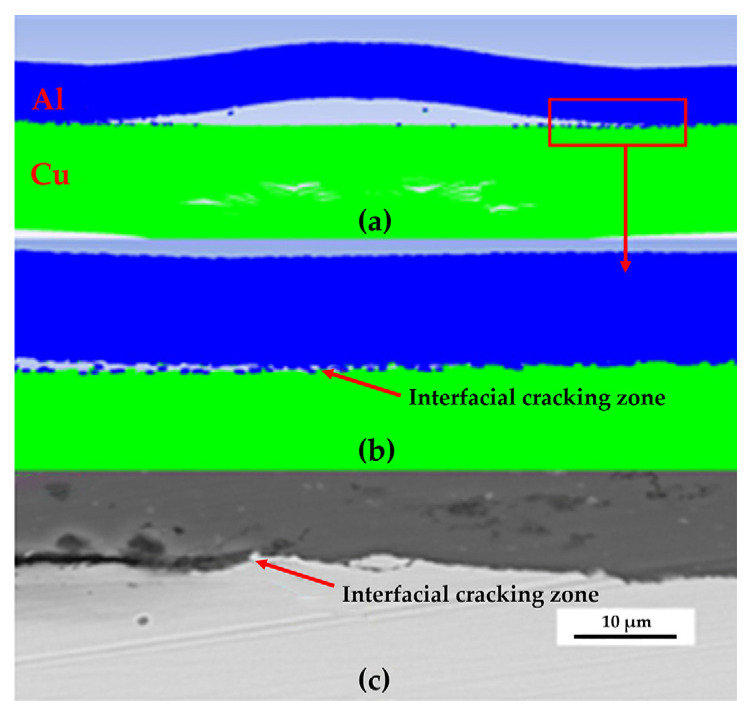
Welding interface morphology and rebound and cracking phenomenon: (**a**) Simulation result diagram; (**b**) Magnified view of the simulation result; (**c**) Microscopic image of the experimental result.

As the laser energy increases, the impact collision velocity correspondingly rises, leading to a transition in the welded bonding interface morphology from a straight profile to a slightly wavy and finally to a distinctly wavy pattern, as illustrated in Figure 10. This phenomenon occurs because the increase in impact velocity results in a higher effective plastic strain at the bonding interface. Consequently, the degree of plastic deformation in both the base plate and the flyer plate intensifies, which in turn promotes an increase in both the wavelength and amplitude of the interfacial waves.

## 5. Non-Equilibrium Mechanical Principles of Irregular Interfaces

This section presents a non-equilibrium mechanical framework to interpret the formation of irregular welding interfaces observed in experiments. The theory links the acceleration field induced by detonation waves to the interfacial morphology, providing a physical basis for understanding the wavy patterns and rebound phenomena described in Section 3 and Section 4.

**Figure 11:**
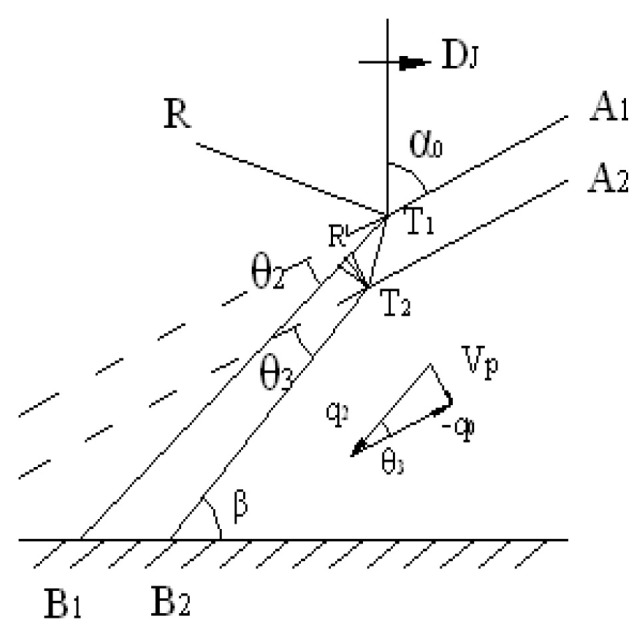
Interaction between detonation and shell.

The motion of the flyer plate driven by explosion in EXW can be described by the theory of interaction between detonation waves and metal surfaces [16]. The specific motion parameters are represented as shown in Figure 11. The detonation wave acting on the flyer surface imparts a deflection angle *θ*_2_ to the interface and simultaneously transmits a refraction wave *T*_1_*T*_2_. When the refraction wave reflects at the lower surface of the flyer, it imparts a deflection angle *θ*_3_ to the free surface and reflects a rarefaction wave *T*_2_ at point *R*′. This rarefaction wave interacts with surface *T*_1_*B*_1_ and gradually causes it to assume the same deflection angle *θ*_3_.

As shown in Figure 11, the refracted wave *T*_1_*T*_2_ is generally not perpendicular or aligned with the lower surface of the flyer. Therefore, its effect can be decomposed into components along and perpendicular to the lower flyer surface, driving the lower surface to produce normal acceleration *a*_n_ and tangential acceleration *a*_τ_.

**Figure 12:**
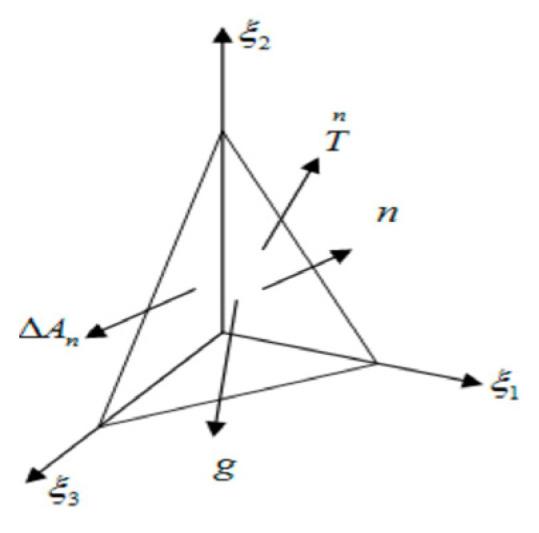
Force analysis of elements.

From Figure 11, it is evident that during EXW, both the surface and interior of the flyer plate are in a non-equilibrium state with intense acceleration characteristics. Accordingly, consider the surface element Δ*A*_n_ shown in Figure 12 and establish the non-equilibrium equation of motion [17,18]. Its unit normal vector is $\vec{n}$, and the surface force per unit area is $\overset{n}{T}$. Let Δ*A*_n_ coincide with the inclined face of a tetrahedral element, while the other three faces are parallel to the coordinate axes *ε*_i_. Due to the acceleration characteristics, inertial and body forces are represented by Δ*mü* and Δ*mg*_i_ (i = 1, 2, 3), respectively, with the dot denoting differentiation with respect to time. On the three coordinate-parallel surfaces Δ*A*_n_Δ*A*_j_ = Δ*A*_n_*n*_j_, nine stress components *σ*_ij_ (where i, j = 1, 2, 3) act, with *n*_j_ = (1, 2, 3) representing the components of the unit normal vector $\vec{n}$. Using the summation convention for repeated indices, the force balance condition can be expressed as

(1) $${\overset{n}{T}}_{i}\Delta A_{n}+\Delta mg_{i}=\sigma_{ij}n_{j}\Delta A_{n}+\Delta m{\ddot{u}}_{i},i=1,2,3$$

Letting *ρ* represent the material mass density and Δm = *ρ*Δ*V*, the above equation can be rewritten as

(2) $${\overset{n}{T}}_{i}=\sigma_{ij}+\rho ({\ddot{u}}_{i}-g_{i})\gamma_{i},i=1,2,3$$

where *γ*_i_ = Δ*V*_n_/Δ*A*_n_.

From Equation (2), it can be concluded that whenever a material surface possesses acceleration distinct from the body force (gravitational acceleration in a gravity field), i.e., *ü*_i_ ≠ *g*_i_, the rate of volume change relative to surface area *γ*_i_ ≠ 0 must be non-zero. As shown in Figure 11, the action of the detonation wave in EXW inevitably imparts significant acceleration to the flyer plate propelling it towards the base plate, thus *ü*_i_ ≠ *g*_i_, leading to *γ*_i_ ≠ 0. A non-zero rate of volume change relative to surface area fundamentally alters the boundary conditions and stress transformation relations, causing the surface force acting on element Δ*A* to no longer be independent of the body force proportional to mass Δ*V* in Δ*m*.

Furthermore, the driven energy of the element can be derived from impulse and acceleration:

(3) $$W=\int Fdx=\int mv\frac{dv}{dx}dx=\int I_{d}{\ddot{u}}_{i}dt$$

Equation (3) describes the driven energy of material element Δ*V* determined by impulse, acceleration, and action time. Differences in this energy among various regions result in diverse welding interface structures. Features such as wavy, curved, or even embedded characteristics on cross-sections and longitudinal profiles are manifestations of the time-integrated results of Equations (2) and (3) in three directions.

## 6. Conclusions

This study presents an approach to laser-controlled energetic material explosion-driven precision welding of microscale metallic foils, investigating the surface morphology, interfacial wave structure, and microstructure of the resulting welds. The key findings are summarized as follows:

(1) A laser-controlled energetic material-driven EXW system for microscale metallic foils was established. By adjusting laser energy and matching energetic working media, laser-controlled energetic material explosion-driven precision welding of microscale metallic foils was achieved.
(2) The correspondence between the welding interface morphological characteristics and laser-controlled energy was observed and presented, and the formation mechanism of the interface characteristics was discussed.
(3) Numerical simulations based on the SPH method successfully reproduced the dynamic welding process, providing insights into the formation of wavy interfaces, rebound, and cracking zones. The non-equilibrium mechanical theory offered a physical interpretation linking the acceleration field to interfacial morphology.

The results presented in this work provide a valuable reference for subsequently optimizing the laser-controlled energetic material explosion-driven welding process for microscale metallic foils.

## Figures and Tables

**Table 1:** Technical parameters table of Hercules-1000.

Laser Parameter	Parameter Value
Wave length (nm)	1064
Pulse Width (ns)	9.480
Output Beam Diameter (mm)	9.81
Pulse Repetition Rate (Hz)	10
Laser Energy (mJ)	52.7–1280

**Table 2:** Experimental parameters.

Experimental Parameter	Parameter Value
Flyer/Base Plate Flyer Dimensions (mm) Base Plate Dimensions (mm)	Industrial Pure Al/T2 Copper 25 × 8 × 0.05 20 × 20 × 0.1
Flyer-Base Gap (mm)	0.2

**Table 3:** Welding results under different ablation layer materials and laser energies.

	Laser Energy Ablation Layer	717 mJ	920 mJ	1280 mJ
Ablation Layer				
Blank Group		×	×	×
Black Paint		√	√	√
Diesel-ammonium Nitrate		×	×	√

Note: In Table 3, √ represents the completion of the welding; × represents the incompletion of the welding.

## Data Availability

The original contributions presented in this study are included in the article. Further inquiries can be directed to the corresponding author.

## Abbreviations

The following abbreviations are used in this manuscript:

MEMS	Micro-electromechanical systems
EXW	Explosive welding
LIW	Laser impact welding
SEM	Scanning electron microscopy
EDX	energy-dispersive X-ray spectroscopy
SPH	Smoothed particle hydrodynamics
